# Peptides in Bronchoalveolar Lavage in Chronic Obstructive Pulmonary Disease

**DOI:** 10.1371/journal.pone.0155724

**Published:** 2016-05-26

**Authors:** Chris H. Wendt, Gary Nelsestuen, Stephen Harvey, Makedonka Gulcev, Matthew Stone, Cavan Reilly

**Affiliations:** 1 Department of Medicine, VA Medical Center, Minneapolis, MN, 55417, United States of America; 2 Department of Medicine, University of Minnesota, Minneapolis, MN, 55455, United States of America; 3 Department of Biochemistry, Molecular Biology and Biophysics, University of Minnesota, Minneapolis, MN, 55455, United States of America; 4 Division of Biostatistics, School of Public Health, University of Minnesota, Minneapolis, MN, 55455, United States of America; University of Alabama-Birmingham, UNITED STATES

## Abstract

**Background:**

Chronic Obstructive Pulmonary Disease (COPD) is a heterogeneous disease with a significant public health burden. Currently there is no biomarker that identifies those at risk of developing COPD, progression of disease or disease phenotypes. We performed metabolomic profiling of bronchoalveolar lavage fluid (BALF) from COPD patients to determine if metabolites correlated with clinical measurements such as lung function, functional status and degree of emphysema.

**Methods:**

Metabolomic components of BALF from 59 subjects with COPD and 20 healthy controls were separated by reversed-phase UPLC and analyzed by ESI-ToF mass spectrometry. We used univariate analysis and multiple regression models to investigate associations between metabolomic features and various clinical variables, such as lung function, functional status as measured by the St. George Respiratory Quotient Score and emphysema as measured by the CT density mask score.

**Results:**

We identified over 3900 features by mass spectrometry, many consistent with peptides. Subjects with severe COPD had increased concentration of peptides compared to controls (p < 9.526e-05). The peptide concentration correlated with spirometry, specifically pulmonary function tests associated with airflow obstruction. There was no correlation with CT density, i.e. emphysema, or functional status.

**Conclusions:**

Metabolomic profiling of BALF in COPD patients demonstrated a significant increase in peptides compared to healthy controls that associated strongly to lung function, but not emphysema or functional status.

## Introduction

Chronic Obstructive Pulmonary Disease (COPD) is currently the third leading cause of death in the U.S., with an economic burden reported in 2007 of $42.6 billion in health care costs and lost productivity. Worldwide it is one of the most prevalent lung diseases causing significant morbidity and mortality [1, 2]. COPD is a complex disease that results from multiple genetic and environmental factors. Despite its long history many questions exist regarding susceptibility, mechanism of disease, and risk factors for the development and progression of disease. It remains unknown why only a subset of smokers develops COPD and which COPD patients are at risk for increased morbidity due to rapid decline in lung function and/or frequent exacerbations.

Historically COPD has been classified as either the clinical diagnosis of chronic bronchitis or the pathological diagnosis of emphysema. Significant overlap exists between these two classifications with 25% of those with COPD developing emphysematous changes [3]. Therefore it is apparent that COPD is a heterogeneous disease in terms of its pathogenesis, clinical presentation and pathophysiology. One current COPD classification scheme is based on severity of disease as defined by limitation of airflow (FEV1), such as in the Global Initiative for Obstructive Lung Disease classification scale[4]. This scale has been used to stratify patients in regards to treatment recommendations, however disease severity is itself not a specific phenotype [5].

In this study we profiled the metabolome of BALF in participants enrolled in the NIH-sponsored Feasibility of Retinoids for the Treatment of Emphysema trial (FORTE) [6, 7]. The participants were clinically stable with moderate to severe COPD with varying degrees of emphysema as measured by CT scan. Statistical modeling was used to identify analytes of the metabolomic profile that associated with clinical parameters such as lung function, emphysema and/or functional status.

## Materials and Methods

### Study Population

Subjects were individuals with COPD and emphysema from the FORTE study [6]. The FORTE study consisted of subjects that underwent bronchoscopy with the collection of bronchoalveolar lavage fluid at baseline (prior to treatment) and again at 3 months after initiation of treatment with drug or placebo[7]. Available for this study were 59 baseline samples (before study drug treatment) from those individuals. Controls consisted of BAL fluid from 20 healthy individuals (10 smokers, 10 non-smokers) that ranged in age from 21–48 from another study. The BALF was performed by instilling 180–240 mL (COPD subjects) or 100–150 mL (controls) of sterile saline solution into the medial or lateral segment of the right middle lobe, followed by aspiration with a target goal of 60 mL of return. All FORTE subjects were former smokers. The clinical characteristics of the sample population are shown in Table 1. Clinical data available for association studies included for controls and COPD subjects: pulmonary function tests; and for COPD subjects: CT density mask score (estimation of amount of emphysema) [6] and the St. George Respiratory Quotient Score (SGRQ). To succinctly refer to the various clinical variables used for the analysis we introduce the following abbreviations: FEV for forced expiratory volume in 1 second, FVC for forced vital capacity, preFEV for FEV before challenge with a bronchodilator and postFEV defined analogously with a bronchodilator and preFEVPP for the percent of preFEV that is predicted based on height, sex and age. Using this notation, the 12 clinical variables used in this analysis were: preFEV:FVC ratio, preFEVPP, postFEVPP, preFEV, postFEV, preFVCPP, postFVCPP, preFVC, postFVC, diffusing capacity of the lung for carbon monoxide (DLCO). All subjects were consented for the parent FORTE study and samples were de-identified for use in this study[6, 7]. This study was deemed research exempt category 4 by the Institutional Review Board of the University of Minnesota (IRB No. 0202M17621 and 0601E80869, PI C. Wendt).

**Table 1:** Subject demographics.

	Study Cohort	Controls	Controls
	N = 59	Smokers	Non-Smokers
		N = 10	N = 10
	Mean + Std	Mean + Std	Mean + Std
Age at time of randomization	66.17 ± 7.29	35 ± 11	37 ± 11
Gender, % male	0.51	0.60	0.40
St. George Score	37.96 ± 14.02	NA	NA
Post-BD %Pred FEV1	46.55 ± 13.85	*	*
Post-BD %Pred FVC	81.05 ± 14.25	*	*
%Pred DLCO	39.33 ± 11.39	NA	NA
%Moderate, GOLD Criteria	34.0	0	0
%Severe/Very Severe GOLD Criteria	66.0	0	0
CT Score, %emphysema	34.65 ± 11.89	NA	NA

*FEV1 and FVC of controls were all >80%. The samples were de-identified and individual spirometry was not available.

### Sample Preparation and Initial LC-MS Analysis

The samples consisted of cell-free BALF stored at –80°C. The samples underwent one thaw at the time of metabolomic analysis and were kept on ice during processing. BAL samples (0.50mL) were prepared by removing protein through the use of disposable C18 reverse-phase resin spin column (MacroSpin, C18, The Nest Group Inc.). Columns were initially conditioned with 500μL acetonitrile (ACN) followed by 0.5 mL water with centrifugation (4 minutes, 2000xg) each time. Samples were acidified to pH 2 with formic acid and applied to the conditioned column. The columns were rinsed twice with 0.4 mL of water/ACN/formic acid (95/5/0.1). The sample was eluted first with 200μL of water/ACN/formic acid (50/50/0.1) followed by 0.2 mL of water/ACN/formic acid (10/90/0.1). The combined 0.4 mL was concentrated by vacuum centrifugation to approximately 0.05 mL. Samples were brought up to 0.1 mL with water/ACN/formic acid (95/5/0.1).

### Multiply Charged Metabolites and Peptides

Many characteristics, such as elution times and the presence of multiply charged analytes (accurate mass and retention time pair) suggested that some of these metabolites were peptides and that they were more abundant in the COPD BALF samples compared to controls. We measured the sum of all peak intensities of multiply charged metabolites that eluted between 1–3.5 minutes as an estimation of peptide concentration. To identify peptides we evaluated three pooled samples: healthy non-smokers, healthy smokers and COPD. Aliquots (0.02 mL) from each sample in each of three groups (10 healthy non-smoker controls, 10 healthy smoker controls and 59 COPD subjects) were pooled for further analysis and identification of the peptides. Ultra Performance Liquid Chromatography (elution as above) was used without mass spectrometry and fractions were collected at 0.5 minute intervals (0.25 mL) between 1.0 and 3.5 minutes.

### Capillary LC-MS/MS

Each fraction was submitted for MS/MS analysis in the orbitrap mass spectrometer for peptide identification. Sample loading, HPLC, and mass spectrometry were performed as previously described [8] with a few exceptions. Electrospray mass spectrometry was performed using a LTQ-Orbitrap XL (ThermoScientific) with spray voltage set to 1.95 kV. Charge state screening was enabled so that undetermined charge states were excluded for data dependent fragmentation. Each sample was run in duplicate to enhance coverage. Analysis of the C-terminal residues of the peptide sequences from the pooled samples demonstrated differences between controls and healthy subjects. The C-terminal residues were normalized to the natural amino acid distribution of the human genome.

### Database Searching and Data Processing

Raw mass spectrometric data obtained from Xcalibur software (ThermoScientific) were extracted using ReAdw (Institute of Systems Biology) to generate mzXML files. Data were searched with SEQUEST V27 against a composite database consisting of the NCBI human database V200806 and its reversed complement and common contaminating protein sequences totaling 70711 entities. A total of 42242 spectra were searched from the control dataset and 65124 spectra were searched from the BOS dataset. Search parameters included no enzyme specification, 100 ppm precursor molecular mass tolerance, 0.8 amu fragment ion mass tolerance, a precursor ion mass range from 700–3600 Da and Met oxidation. SEQUEST output was organized and peptide probabilities were calculated through Peptide Prophet using Scaffold (Proteome Software, Inc., Portland, OR). Peptide identifications were filtered using the following parameters: 95% peptide probability and 7 ppm for precursor mass tolerance. Estimated false positive rates were calculated from identified spectra using the equation: (2 x reverse database identifications/(forward + reverse database identifications)*100). Peptide false discovery rates were determined using the following calculation: (reverse database identifications/(forward + reverse database identifications)*100). All identified MS/MS spectra are listed in S1 Table and can be downloaded from Figshare (*figshare*.*com*) with the following https://figshare.com/s/fbd31d78b849d45e5bdf.

### Statistical Analysis

To investigate associations between identified peptides and the clinical variables we first examined the extent of univariate associations between each clinical variable and all of the detected analytes. To this end, linear regression models were fit with the logarithm of the analyte intensity as the response variable and sex, age and one of 12 clinical variables as explanatory variables (sex and age were included as they are potential confounders since they are both known to be associated with lung function test results and are potentially related to analyte levels). Since the analyte intensities can be equal to 0, the number 1 was added to all analyte levels prior to taking the log. Following this, we fit multiple regression models with all 12 clinical variables present (in addition to sex and age). We then tested for the joint effect of all 12 clinical variables on the analyte intensities using an *F*-test based on ratios of sums of squares from regression models that include all 12 clinical variables, sex and age to regression models that just include sex and age. While the linear regression models for assessing if there are univariate associations between analyte levels and our clinical data can determine if analyte levels are associated with the clinical data at the level of each variable, the test for the joint effect can be interpreted as testing the more global association between pulmonary function testing and analyte levels.

## Results

### Total Metabolite Analytes in COPD BALF versus Healthy Controls

**Fig 1:**
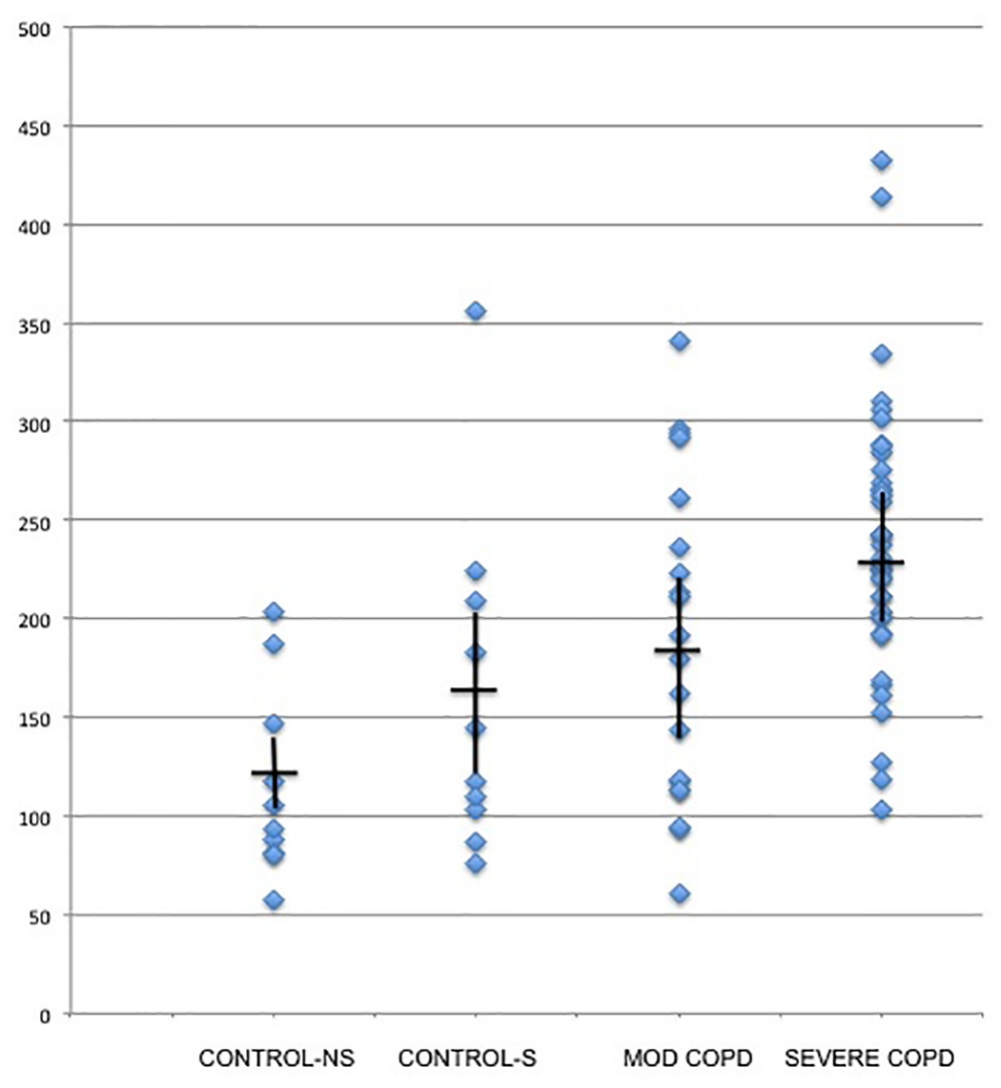
Peptide analyte intensities of BALF samples. *p =* 9.526e-05 (Kruskal-Wallis) for differences among the groups. Moderate COPD (GOLD 2), Severe COPD (GOLD 3–4)

We detected over 3900 analytes in the BAL fluid by LC-mass spectrometry of the 59 COPD subjects and 20 controls. Circumstantial factors, such as short elution times (< 3.5 minutes) and the presence of multiply charged analytes, suggested that 690 of these analytes from our LC-MS analysis were peptides that ranged in size from 171.17 to 995.56 m/z. These peptide analytes are the basis of this study. Currently there is no accepted methodology to quantify metabolites detected by mass spectrometry. As an estimate of peptide concentration we measured the sum of all peak intensities detected by the LCT mass spectrometer for each individual sample that were consistent with being a peptide, i.e the analytes were multiply charged with an elution time < 3.5 from the UPLC (Fig 1). We did not correct for dilution effects that may have occurred due to varying amounts of BAL fluid instilled at the time of specimen collection. It was apparent that there was an increase in total peak intensity in samples from those with moderate (GOLD 2) or severe COPD (GOLD 3–4) compared to healthy controls, both smokers and non-smokers. We used the Kruskal-Wallis test to look for differences among the groups regarding the total number of peaks. This revealed a *p-*value of 9.526e-05 that was mainly driven by differences between the control non-smokers and the severe COPD group.

### Associations between Clinical Variables and Peptide Analytes in COPD Patients

To identify the BALF peptides we collected fractions from the UPLC chromatogram (10 Control-smokers, 10 Control-nonsmokers, 59 COPD-moderate+severe) corresponding to the retention time expected for peptides. Due to the small volume from these UPLC fractions, samples were pooled in their respective groups for peptide analysis. The collected fractions were analyzed in an LTQ-Orbitrap XL mass spectrometer (ThermoScientific). Peptide identifications were stringently filtered to give an estimated false positive rate of 0.16%, corresponding to a protein false discovery rate of 1.2%. Overall, the COPD samples had increased evidence of protease activity due to the comparatively high amount of peptides identified. To identify the proteases involved in generating the peptides, we performed analysis of the C-terminal residues of the identified peptide sequences. These C-terminal amino acids were tabulated and normalized to the natural amino acid distribution of the human genome. Shown in Table 2 are the results for the control nonsmokers and COPD subjects. Overall the frequency was lower in the non-smoker controls consistent with a lower concentration of peptides. The peptides from the control non-smoker samples had the highest occurrence of the following C-terminal residues F, L, Q, R, W and Y. Those with COPD were similar to non-smoker subjects except W was not increased and they demonstrated elevated C-terminal frequencies of V and A. The latter cleavage sites, valine (V) and alanine (A), are consistent with cleavage by elastase 2.

**Table 2:** C-Terminal Peptide Analysis.

c-terminal residue	frequency	% frequency	% of frequency normalized to naturally occurring AA frequency	frequency	% frequency	% of frequency normalized to naturally occurring AA frequency
	Controls-Nonsmokers			COPD-Severe + Moderate		
**A**	0	0	0	21	12.3	**1.7**
C	0	0	0	1	0.6	0.2
D	0	0	0	4	2.3	0.4
E	0	0	0	1	0.6	0.1
F	2	9.5	2.4	17	9.9	2.5
G	1	4.8	0.6	1	0.6	0.1
H	0	0	0	2	1.2	0.4
**I**	0	0	0	2	1.2	0.3
K	0	0	0	8	4.7	0.6
L	2	9.5	1.3	25	14.6	1.9
M	0	0	0	9	5.3	2.9
N	0	0	0	3	1.8	0.4
P	0	0	0	4	2.3	0.5
Q	3	14.3	3.9	13	7.6	2.1
R	2	19.0	4.5	23	13.4	3.2
S	0	0	0	10	5.8	0.7
**T**	0	0	0	5	2.9	0.5
**V**	0	0	0	13	7.6	1.1
W	0	4.76	3.7	2	1.2	0.9
Y	8	38.1	11.5	7	4.1	1.2
TOTAL	21	100		171	100	

**Fig 2:**
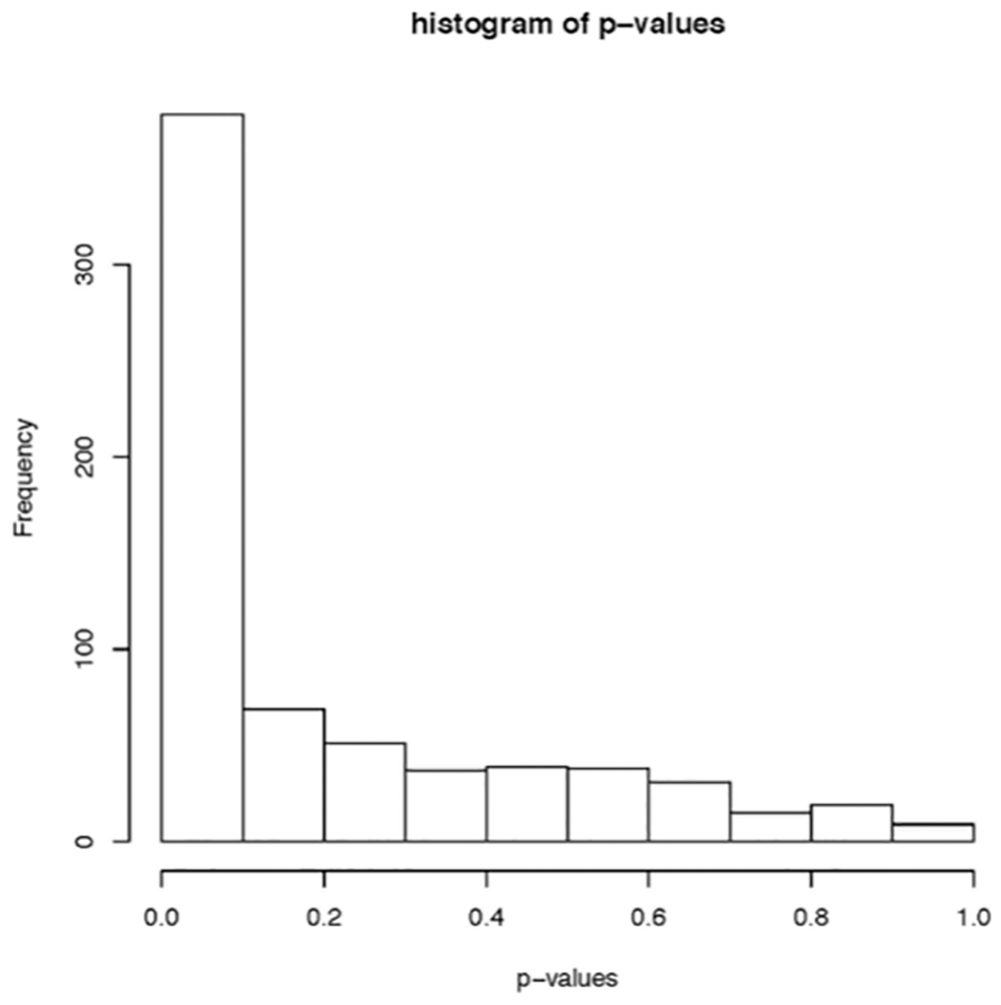
Histogram of *p-*values. A histogram displaying the distribution of *p*-values for jointly testing for an association between the clinical variables used to assess lung function and the levels of the analytes in the peptide profile. The large number of small *p*-values indicates that there is an association between the clinical data set and the peptide analytes in BALF.

**Fig 3:**
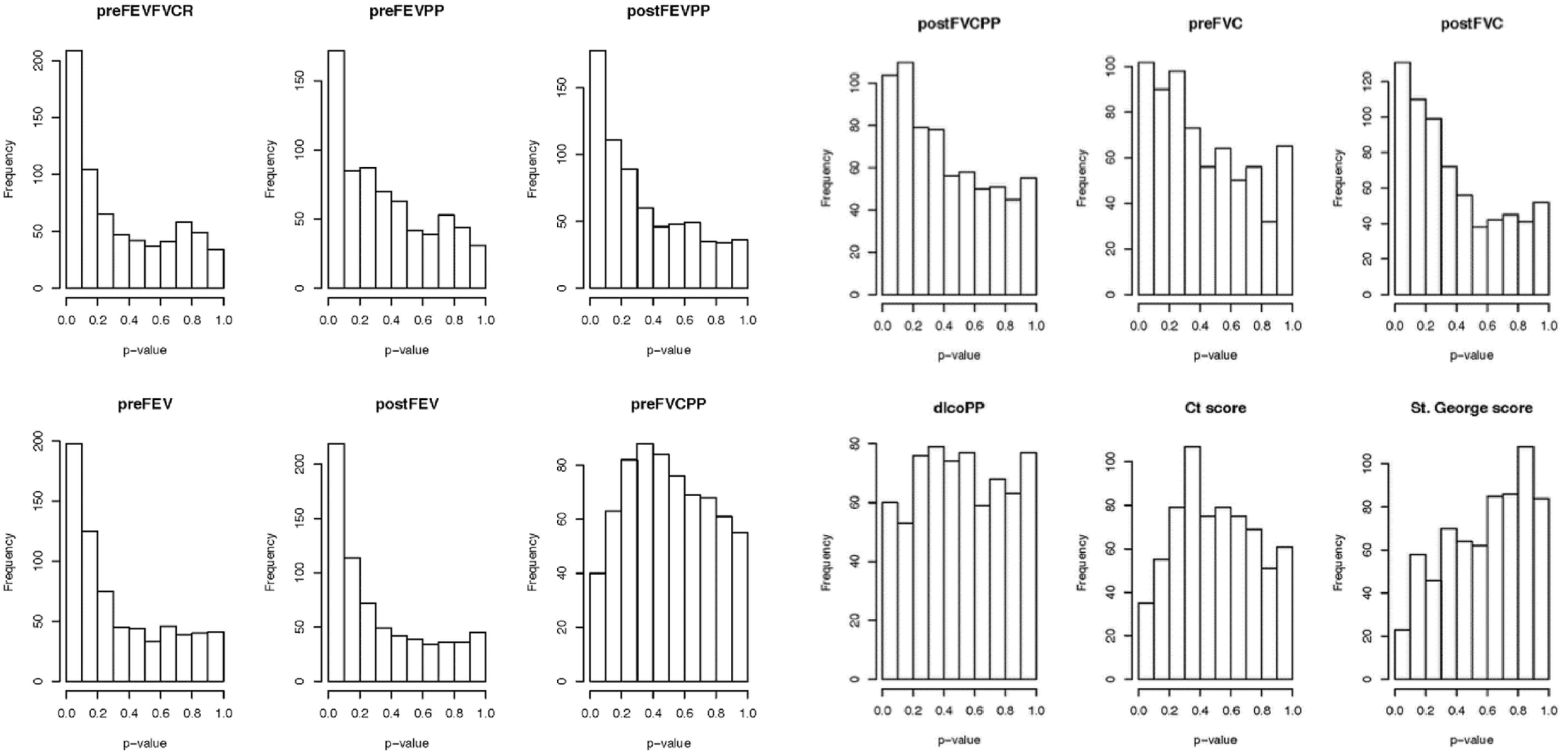
Histograms of *p-*values for individual tests. Histograms of *p*-values for testing for an association between the identified peptide analytes and twelve clinical variables that are used to assess lung function, degree of emphysema and functional status.

To determine if peptide quantity correlated with a specific COPD phenotype we investigated the relationship between our clinical data and the peptide analyte intensities from individual samples via linear models that included gender, age and each of the lung function tests for the COPD patients. Our initial analysis demonstrated a significant association between the clinical variables relating to lung function (spirometry, DLCOPP, CT density and SGRQ) and the levels of the analytes in the peptide profile (Fig 2). To determine which COPD characteristic associated with peptide levels we evaluated the individual clinical variable associations (Fig 3) in the form of *p*-value histograms. The peptide analytes demonstrated an overall surplus of small *p*-values to all of the pulmonary spirometric tests with the exception of preFVCPP. Specifically, the pulmonary function tests associated with airflow obstruction (FEV1 and FEVFVCR) had the smallest *p-*values. Whereas, measures correlating to emphysema, such as CT density mask and DLCOPP, did not correlate to peptide analyte intensity. The SGRQ score, a measurement of functional status, also did not demonstrate an association.

## Discussion

We found that many of the analytes from the LC-MS data of COPD BALF were suggestive of peptides due to their multiply charged state and retention times. Analysis of data from the LTQ-orbitrap XL confirmed increased peptides in the COPD samples relative to controls. To classify the proteases involved in the generation of these peptides we identified the peptides’ C-terminal amino acids. The increased prevalence of valine (V) and alanine (A)at the C-terminal position suggested that some of the peptides were generated by elastase-2 activity in the COPD samples. Increased protease activity and protease-antiprotease imbalance in COPD is well described[9–11]. Several families of proteinases, including serine proteinases, metalloproteinases and cysteine proteinases, have been implicated in COPD[12]. Initial early studies focused on identifying elastase generated peptides by ELISA in COPD[13, 14]. However, subsequent studies concentrated mainly on characterizing the various protease types with less emphasis on down-stream products[15, 16]. Many have assumed that increased protease activity is responsible for the emphysematous COPD phenotype as *in vivo* models of excess proteases create an emphysema model of COPD[17]. In our study we identified increased peptides in COPD BALF and sought to identify the corresponding COPD phenotype that associated with this elevation in protease activity. To identify the COPD phenotype with increased peptide levels we sought associations of the peptide analytes with clinical features within the COPD subjects. With multiple clinical characteristics for possible biomarker association, the statistical analysis approach is key to avoid false negatives. We used linear statistical models to determine the relationship between our clinical data and the peptide analytes in the COPD samples. A common presentation of this type of data is in the form of *p*-value histograms [18]. The expected shape of the p-value histogram that reflects strong associations among the variables is one where most values are close to zero and the frequency decreases as the *p*-value increases. From this we see a strong association between the peptide analyte intensity and several of our clinical variables.

In our first linear model approach the *p*-value histograms demonstrated a high association of peptide analytes to pulmonary spirometric tests with the exception of preFVC percent predicted. This suggests that airflow obstruction, as demonstrated by FEV1 and FEV1/FVC ratio, has a strong association with the peptide analytes. In a separate statistical approach we conducted hierarchical cluster analysis of the various analytes to identify COPD subgroups based on metabolomic profiles (data not shown). Once clusters were identified we tested whether our 11 clinical variables differed between 2 prominent clusters. Again, the most significant clinical variables that associated with the clusters were pulmonary function, especially the FEV1, which is a measure of airflow limitation. Interestingly, the peptide analytes did not correlate to indicators of the degree of emphysema such as DLCOPP and CT scan density or functional status as determined by the SGRQ. It should be noted that all subjects with COPD had varying degrees of emphysema as measured by CT scan density and DLCO and the CT density scores did not correlate to airflow obstruction (data not shown).

There are several reasons why lung function and airflow limitation may associate with peptide abundance in the BALF. When performing bronchial lavage, both the alveolar structure and distal airways are sampled. These distal conducting airways are a site of airflow obstruction in COPD[19]. Peptides are a direct result of proteolysis and our C-terminal sequence analysis supports that some of the proteolytic activity is from elastase, a product of neutrophils[20, 21]. Elevated peptides in BALF have been associated with smoking and COPD[22, 23]. An increased inflammatory state and/or bacterial burden would create a proteolytic rich environment that results in structural damage of the distal airways, airflow obstruction and changes in the local metabolome as seen in our study. These peptides themselves may also be pro-inflammatory and contribute to the cycle of inflammation and worsening airflow obstruction[23, 24]. Measurement of neutrophils and proteases directly have been reported in COPD, however, increased levels do not necessarily indicate increased activity. Therefore, BALF peptide measurements may function as a biomarker for increased protease activity and could also function as a therapeutic target.

Limitations of this study include age as a potential confounder since the controls were not age-matched to the COPD patients and the smoker control subjects had a lower pack year history of smoking (mean 17.5 vs. 59 pack years). We are not aware of marked increase in peptides in BALF in aging, but cannot exclude the possibility that age could be a contributing factor. Another limitation is the need for an invasive test, bronchoscopy with bronchoalveolar lavage, to measure the peptides. Future studies measuring peptides in induced sputum could alleviate the need for bronchoscopy and provide a safe and easily obtained diagnostic biological sample to test whether this biomarker is useful in identifying those at risk of rapid decline in lung function

## Conclusion

In summary, we report increased peptides in BALF from COPD patients and peptide sequencing reveals many are consistent with elastase-2 activity. This increase in peptides correlate to airflow obstruction, and not to emphysema, suggesting they are a biomarker for airflow obstruction.

## Supporting Information

S1 Table Features at 1% and 5% FDR. (DOCX)
