# Supplementary material for: Peptides in Bronchoalveolar Lavage in Chronic Obstructive Pulmonary Disease
Source: PLoS One. 2016 May 26;11(5):e0155724. doi: 10.1371/journal.pone.0155724 (PMC4881978; doi:10.1371/journal.pone.0155724)
Supplement: S1 Table — (DOCX) [file pone.0155724.s001.docx]

S1 Table. Features at 1% and 5 % FDR

Features at 1%

| Retention Times | m/z value |
| --- | --- |
| 2.1427 | 344.6909 |
| 2.1982 | 534.3449 |
| 2.3201 | 849.4434 |
| 2.3213 | 833.4715 |
| 2.3218 | 828.5171 |
| 2.435 | 655.3502 |
| 2.4972 | 537.3405 |
| 2.6624 | 604.3305 |
| 2.6724 | 831.4565 |
| 2.7025 | 881.4465 |
| 2.716 | 875.4821 |
| 2.7246 | 326.0914 |
| 2.7455 | 925.4746 |
| 2.9314 | 448.3163 |
| 3.1028 | 462.3324 |
| 3.2018 | 506.2287 |
| 3.6039 | 426.3118 |
| 4.7564 | 419.3297 |
| 6.1611 | 358.2048 |
| 6.3103 | 324.225 |

Features at 5% FDR

| Retention Times | m/z value |
| --- | --- |
| 1.4354 | 209.1448 |
| 1.4581 | 875.2337 |
| 1.4826 | 237.1369 |
| 1.5008 | 975.4894 |
| 1.6406 | 488.3082 |
| 1.6418 | 379.1969 |
| 1.642 | 478.1856 |
| 1.6495 | 500.61 |
| 1.667 | 336.2695 |
| 1.6883 | 423.2213 |
| 1.6891 | 418.2643 |
| 1.6905 | 227.1905 |
| 1.6913 | 281.1948 |
| 1.6913 | 450.2306 |
| 1.6917 | 453.3424 |
| 1.7138 | 437.1971 |
| 1.7149 | 432.2383 |
| 1.7168 | 768.8448 |
| 1.7259 | 207.1637 |
| 1.7271 | 413.2916 |
| 1.7293 | 270.1703 |
| 1.7322 | 738.3309 |
| 1.733 | 467.2467 |
| 1.7332 | 462.2897 |
| 1.7356 | 489.2972 |
| 1.7711 | 579.3123 |
| 1.7745 | 656.3444 |
| 1.7806 | 511.27 |
| 1.7815 | 506.3157 |
| 1.8071 | 552.2894 |
| 1.8102 | 808.8209 |
| 1.8256 | 451.2158 |
| 1.8264 | 429.2352 |
| 1.8292 | 787.4794 |
| 1.8301 | 553.3063 |
| 1.8301 | 637.2673 |
| 1.8304 | 621.294 |
| 1.8311 | 257.1769 |
| 1.834 | 298.1968 |
| 1.8354 | 446.2581 |
| 1.8375 | 683.0983 |
| 1.8383 | 843.6162 |
| 1.8393 | 675.0942 |
| 1.8416 | 582.3098 |
| 1.8563 | 570.2604 |
| 1.8906 | 517.2276 |
| 1.8944 | 599.3245 |
| 1.8954 | 594.3694 |
| 1.9033 | 626.3351 |
| 1.9135 | 490.288 |
| 1.9491 | 377.1718 |
| 1.9509 | 643.3501 |
| 1.9519 | 638.3949 |
| 1.9534 | 670.3605 |
| 1.9559 | 539.2662 |
| 1.9579 | 534.3113 |
| 1.9593 | 665.4038 |
| 1.9673 | 394.1816 |
| 1.9788 | 679.4241 |
| 1.9799 | 475.3305 |
| 2.0092 | 372.2376 |
| 2.0099 | 682.4208 |
| 2.0111 | 352.7055 |
| 2.0121 | 687.376 |
| 2.0121 | 714.3853 |
| 2.0153 | 336.2691 |
| 2.0212 | 578.3374 |
| 2.0212 | 583.2932 |
| 2.0337 | 453.3424 |
| 2.0528 | 416.2531 |
| 2.0622 | 701.3519 |
| 2.0649 | 731.4027 |
| 2.0818 | 649.3013 |
| 2.0868 | 622.3632 |
| 2.0878 | 627.3203 |
| 2.1039 | 441.1885 |
| 2.1055 | 719.3411 |
| 2.1172 | 775.4306 |
| 2.1173 | 776.4321 |
| 2.1175 | 396.7311 |
| 2.1176 | 797.4848 |
| 2.1177 | 770.4757 |
| 2.1178 | 802.4387 |
| 2.1416 | 354.2453 |
| 2.1427 | 344.6909 |
| 2.1442 | 671.3467 |
| 2.1444 | 649.3638 |
| 2.1529 | 348.7228 |
| 2.1599 | 359.7134 |
| 2.1603 | 701.3915 |
| 2.1637 | 724.469 |
| 2.1642 | 263.1853 |
| 2.17 | 460.2859 |
| 2.1729 | 814.5009 |
| 2.1742 | 983.5071 |
| 2.1754 | 293.1903 |
| 2.1785 | 841.5128 |
| 2.1821 | 480.2567 |
| 2.1855 | 654.0131 |
| 2.1878 | 449.7506 |
| 2.1915 | 603.2771 |
| 2.1982 | 534.3449 |
| 2.2009 | 693.3893 |
| 2.2012 | 366.7022 |
| 2.2022 | 710.4154 |
| 2.2026 | 715.3718 |
| 2.219 | 370.7352 |
| 2.2206 | 381.7266 |
| 2.2217 | 740.462 |
| 2.2221 | 438.2766 |
| 2.2233 | 440.7535 |
| 2.2238 | 858.5247 |
| 2.2457 | 524.2793 |
| 2.2574 | 518.3162 |
| 2.265 | 754.4418 |
| 2.2659 | 388.7147 |
| 2.2675 | 523.2746 |
| 2.2683 | 578.3731 |
| 2.2697 | 902.553 |
| 2.271 | 907.5079 |
| 2.2712 | 403.7383 |
| 2.2712 | 767.4605 |
| 2.2957 | 322.2528 |
| 2.3125 | 533.37 |
| 2.3138 | 798.47 |
| 2.3158 | 804.427 |
| 2.3162 | 803.4233 |
| 2.3198 | 951.5345 |
| 2.3199 | 417.0481 |
| 2.32 | 946.5792 |
| 2.3201 | 849.4434 |
| 2.321 | 414.7592 |
| 2.3213 | 833.4715 |
| 2.3216 | 568.308 |
| 2.3218 | 828.5171 |
| 2.322 | 423.2719 |
| 2.322 | 834.4767 |
| 2.3223 | 425.7495 |
| 2.3276 | 622.4037 |
| 2.329 | 376.0233 |
| 2.3644 | 432.7393 |
| 2.3658 | 506.7948 |
| 2.3663 | 842.4955 |
| 2.3668 | 995.5606 |
| 2.3678 | 877.4966 |
| 2.3692 | 436.7718 |
| 2.3696 | 872.543 |
| 2.3704 | 447.7609 |
| 2.387 | 450.2516 |
| 2.3906 | 612.3357 |
| 2.3996 | 611.3255 |
| 2.415 | 922.526 |
| 2.4176 | 921.5246 |
| 2.435 | 655.3502 |
| 2.4412 | 669.8644 |
| 2.4471 | 661.3162 |
| 2.4582 | 981.5237 |
| 2.4583 | 935.5065 |
| 2.46 | 965.5496 |
| 2.4643 | 966.5491 |
| 2.4857 | 516.2822 |
| 2.4861 | 314.1929 |
| 2.494 | 494.2691 |
| 2.4972 | 537.3405 |
| 2.5002 | 759.4352 |
| 2.5017 | 705.3423 |
| 2.5182 | 220.1484 |
| 2.5276 | 287.1843 |
| 2.5339 | 699.3765 |
| 2.5373 | 176.125 |
| 2.5375 | 535.3822 |
| 2.5429 | 867.4377 |
| 2.5474 | 538.2938 |
| 2.5518 | 803.4607 |
| 2.5526 | 551.3474 |
| 2.5563 | 328.2014 |
| 2.5564 | 629.3024 |
| 2.5647 | 749.3677 |
| 2.5657 | 560.3078 |
| 2.5665 | 619.3366 |
| 2.5682 | 249.1717 |
| 2.5723 | 613.3276 |
| 2.5729 | 985.6087 |
| 2.5737 | 743.4015 |
| 2.5884 | 209.145 |
| 2.6084 | 793.3968 |
| 2.6172 | 781.3097 |
| 2.621 | 995.2926 |
| 2.6237 | 787.4278 |
| 2.6588 | 837.4225 |
| 2.6624 | 604.3305 |
| 2.6719 | 337.2302 |
| 2.6724 | 831.4565 |
| 2.6933 | 471.3518 |
| 2.6975 | 73.7956 |
| 2.6981 | 73.6921 |
| 2.7025 | 881.4465 |
| 2.716 | 875.4821 |
| 2.7246 | 326.0914 |
| 2.7331 | 648.3548 |
| 2.7455 | 925.4746 |
| 2.7575 | 919.5081 |
| 2.7597 | 914.5548 |
| 2.799 | 963.5311 |
| 2.8587 | 663.7508 |
| 2.8846 | 561.3621 |
| 2.923 | 421.2128 |
| 2.9314 | 448.3163 |
| 2.9497 | 910.4617 |
| 2.9983 | 279.2154 |
| 3.036 | 435.2866 |
| 3.0645 | 352.249 |
| 3.1012 | 366.2423 |
| 3.1028 | 462.3324 |
| 3.121 | 265.2007 |
| 3.1358 | 406.3094 |
| 3.1603 | 382.2028 |
| 3.2018 | 506.2287 |
| 3.2215 | 396.2643 |
| 3.2387 | 299.1595 |
| 3.2749 | 349.2532 |
| 3.3353 | 383.235 |
| 3.34 | 476.3475 |
| 3.5418 | 419.2899 |
| 3.5904 | 311.2546 |
| 3.6039 | 426.3118 |
| 3.6161 | 384.1805 |
| 3.6347 | 363.2723 |
| 3.71 | 462.2396 |
| 3.822 | 375.3251 |
| 3.9018 | 524.359 |
| 3.903 | 552.2559 |
| 3.9056 | 523.1987 |
| 3.9062 | 659.3124 |
| 3.9065 | 512.2056 |
| 3.9071 | 360.128 |
| 3.9072 | 507.2279 |
| 3.9076 | 179.1016 |
| 3.9087 | 407.2023 |
| 3.9097 | 811.3974 |
| 3.9107 | 194.1334 |
| 3.9123 | 340.219 |
| 4.0569 | 614.414 |
| 4.1585 | 412.2097 |
| 4.4507 | 313.2471 |
| 4.492 | 213.21 |
| 4.7564 | 419.3297 |
| 4.9231 | 679.3958 |
| 5.0309 | 371.3298 |
| 5.0669 | 249.1721 |
| 5.2412 | 359.2446 |
| 5.9804 | 397.2859 |
| 6.1099 | 663.4365 |
| 6.1611 | 358.2048 |
| 6.3103 | 324.225 |
| 6.4477 | 762.5419 |
| 8.0654 | 536.1726 |
| 8.2803 | 265.1995 |
| 8.3096 | 586.3744 |
| 8.3298 | 595.4725 |
| 8.3541 | 628.1956 |
| 8.5118 | 735.4647 |
| 8.6383 | 395.3027 |
| 8.6839 | 478.4053 |
| 9.3556 | 907.2602 |
| 9.4172 | 516.405 |
| 9.8199 | 694.5819 |

Features at 10% FDR

| Retention Times | m/z value |
| --- | --- |
| 1.3245 | 235.1935 |
| 1.4354 | 209.1448 |
| 1.4581 | 875.2337 |
| 1.459 | 861.5774 |
| 1.4596 | 875.0324 |
| 1.4818 | 990.0655 |
| 1.4826 | 237.1369 |
| 1.4832 | 993.7837 |
| 1.4844 | 993.6389 |
| 1.5008 | 975.4894 |
| 1.5009 | 975.3414 |
| 1.5537 | 251.1496 |
| 1.5587 | 792.0684 |
| 1.5973 | 554.4826 |
| 1.6304 | 647.3786 |
| 1.6362 | 548.6725 |
| 1.6406 | 488.3082 |
| 1.6418 | 379.1969 |
| 1.642 | 478.1856 |
| 1.6495 | 500.61 |
| 1.667 | 336.2695 |
| 1.6674 | 543.9396 |
| 1.6743 | 397.2151 |
| 1.6879 | 490.1824 |
| 1.6883 | 423.2213 |
| 1.6889 | 475.6015 |
| 1.6891 | 418.2643 |
| 1.6893 | 712.9054 |
| 1.6905 | 227.1905 |
| 1.6909 | 290.1922 |
| 1.691 | 475.9378 |
| 1.6913 | 281.1948 |
| 1.6913 | 450.2306 |
| 1.6917 | 453.3424 |
| 1.7135 | 358.2108 |
| 1.7138 | 437.1971 |
| 1.7149 | 432.2383 |
| 1.7168 | 768.8448 |
| 1.7259 | 207.1637 |
| 1.7271 | 413.2916 |
| 1.7276 | 248.1894 |
| 1.7293 | 270.1703 |
| 1.7314 | 494.2575 |
| 1.7322 | 738.3309 |
| 1.7325 | 612.32 |
| 1.733 | 467.2467 |
| 1.7332 | 462.2897 |
| 1.7356 | 489.2972 |
| 1.737 | 820.9187 |
| 1.7663 | 402.2347 |
| 1.7666 | 407.191 |
| 1.7711 | 579.3123 |
| 1.7745 | 656.3444 |
| 1.7806 | 511.27 |
| 1.7815 | 506.3157 |
| 1.7834 | 538.2831 |
| 1.8012 | 860.8755 |
| 1.8013 | 861.1288 |
| 1.8029 | 705.7119 |
| 1.8071 | 552.2894 |
| 1.8102 | 808.8209 |
| 1.8256 | 451.2158 |
| 1.8264 | 429.2352 |
| 1.8292 | 787.4794 |
| 1.8301 | 553.3063 |
| 1.8301 | 637.2673 |
| 1.8304 | 621.294 |
| 1.8311 | 257.1769 |
| 1.8338 | 550.343 |
| 1.8339 | 491.3238 |
| 1.834 | 298.1968 |
| 1.8354 | 446.2581 |
| 1.8375 | 683.0983 |
| 1.8383 | 843.6162 |
| 1.8393 | 675.0942 |
| 1.8404 | 843.3679 |
| 1.8416 | 582.3098 |
| 1.8455 | 392.1904 |
| 1.8468 | 872.6225 |
| 1.85 | 577.3473 |
| 1.8558 | 559.322 |
| 1.8563 | 570.2604 |
| 1.869 | 469.35 |
| 1.8906 | 517.2276 |
| 1.8922 | 332.2241 |
| 1.8944 | 599.3245 |
| 1.8954 | 594.3694 |
| 1.9033 | 626.3351 |
| 1.9052 | 634.853 |
| 1.9135 | 490.288 |
| 1.9417 | 674.7358 |
| 1.9458 | 517.282 |
| 1.9491 | 377.1718 |
| 1.9509 | 643.3501 |
| 1.9519 | 638.3949 |
| 1.9534 | 670.3605 |
| 1.9559 | 539.2662 |
| 1.9579 | 534.3113 |
| 1.9593 | 665.4038 |
| 1.9673 | 394.1816 |
| 1.9712 | 832.453 |
| 1.9788 | 679.4241 |
| 1.9799 | 475.3305 |
| 2.0092 | 372.2376 |
| 2.0099 | 682.4208 |
| 2.0111 | 352.7055 |
| 2.0121 | 687.376 |
| 2.0121 | 714.3853 |
| 2.0123 | 709.434 |
| 2.0153 | 336.2691 |
| 2.0212 | 578.3374 |
| 2.0212 | 583.2932 |
| 2.0245 | 703.339 |
| 2.0324 | 548.7775 |
| 2.0337 | 453.3424 |
| 2.0528 | 416.2531 |
| 2.0622 | 701.3519 |
| 2.0633 | 374.7188 |
| 2.0649 | 731.4027 |
| 2.0663 | 726.4459 |
| 2.069 | 753.4575 |
| 2.081 | 485.3475 |
| 2.0818 | 649.3013 |
| 2.0868 | 622.3632 |
| 2.0878 | 627.3203 |
| 2.103 | 329.1913 |
| 2.1039 | 441.1885 |
| 2.1055 | 719.3411 |
| 2.1056 | 673.3409 |
| 2.1172 | 775.4306 |
| 2.1173 | 776.4321 |
| 2.1175 | 396.7311 |
| 2.1176 | 797.4848 |
| 2.1177 | 770.4757 |
| 2.1178 | 802.4387 |
| 2.1294 | 490.3228 |
| 2.1416 | 354.2453 |
| 2.1427 | 344.6909 |
| 2.1442 | 671.3467 |
| 2.1444 | 649.3638 |
| 2.1447 | 624.4963 |
| 2.1489 | 696.4321 |
| 2.1523 | 717.3649 |
| 2.1529 | 348.7228 |
| 2.1575 | 679.4086 |
| 2.1599 | 359.7134 |
| 2.1603 | 701.3915 |
| 2.1609 | 392.7501 |
| 2.1612 | 357.2365 |
| 2.1637 | 724.469 |
| 2.1642 | 263.1853 |
| 2.17 | 460.2859 |
| 2.1729 | 814.5009 |
| 2.1742 | 983.5071 |
| 2.1747 | 537.294 |
| 2.1754 | 293.1903 |
| 2.1777 | 820.461 |
| 2.1779 | 846.4658 |
| 2.1785 | 841.5128 |
| 2.1821 | 480.2567 |
| 2.1855 | 654.0131 |
| 2.1878 | 449.7506 |
| 2.1915 | 603.2771 |
| 2.1982 | 534.3449 |
| 2.2009 | 693.3893 |
| 2.2012 | 366.7022 |
| 2.2022 | 710.4154 |
| 2.2026 | 715.3718 |
| 2.2058 | 561.8031 |
| 2.2081 | 319.2079 |
| 2.2131 | 379.2478 |
| 2.2173 | 504.3103 |
| 2.219 | 370.7352 |
| 2.2206 | 381.7266 |
| 2.2217 | 740.462 |
| 2.2221 | 438.2766 |
| 2.2233 | 440.7535 |
| 2.2236 | 885.5394 |
| 2.2238 | 858.5247 |
| 2.2291 | 450.1661 |
| 2.2445 | 479.2861 |
| 2.2457 | 524.2793 |
| 2.2547 | 647.3068 |
| 2.2574 | 518.3162 |
| 2.2647 | 784.4868 |
| 2.265 | 754.4418 |
| 2.2659 | 388.7147 |
| 2.2675 | 523.2746 |
| 2.2683 | 578.3731 |
| 2.2697 | 902.553 |
| 2.2704 | 929.5595 |
| 2.2706 | 790.4499 |
| 2.271 | 907.5079 |
| 2.2712 | 403.7383 |
| 2.2712 | 767.4605 |
| 2.2725 | 384.2316 |
| 2.2735 | 872.506 |
| 2.2739 | 363.2412 |
| 2.2814 | 653.3408 |
| 2.2839 | 437.281 |
| 2.2957 | 322.2528 |
| 2.2968 | 557.3559 |
| 2.3125 | 533.37 |
| 2.3138 | 798.47 |
| 2.3158 | 804.427 |
| 2.3162 | 803.4233 |
| 2.3183 | 978.5404 |
| 2.319 | 952.537 |
| 2.3194 | 811.4916 |
| 2.3198 | 951.5345 |
| 2.3199 | 417.0481 |
| 2.32 | 946.5792 |
| 2.3201 | 849.4434 |
| 2.3205 | 701.4921 |
| 2.321 | 414.7592 |
| 2.3213 | 573.264 |
| 2.3213 | 833.4715 |
| 2.3216 | 568.308 |
| 2.3218 | 828.5171 |
| 2.322 | 423.2719 |
| 2.322 | 834.4767 |
| 2.3223 | 425.7495 |
| 2.3276 | 605.3738 |
| 2.3276 | 622.4037 |
| 2.329 | 376.0233 |
| 2.3336 | 428.2461 |
| 2.3554 | 760.0644 |
| 2.3644 | 432.7393 |
| 2.3658 | 506.7948 |
| 2.3659 | 990.6019 |
| 2.3663 | 842.4955 |
| 2.3668 | 995.5606 |
| 2.3678 | 877.4966 |
| 2.3692 | 436.7718 |
| 2.3696 | 872.543 |
| 2.3704 | 447.7609 |
| 2.371 | 619.2739 |
| 2.3729 | 893.4718 |
| 2.387 | 450.2516 |
| 2.3906 | 612.3357 |
| 2.3908 | 803.9829 |
| 2.3996 | 611.3255 |
| 2.4025 | 847.4531 |
| 2.4055 | 847.7021 |
| 2.4066 | 678.7633 |
| 2.4082 | 848.2061 |
| 2.415 | 922.526 |
| 2.4161 | 916.5679 |
| 2.4172 | 937.4982 |
| 2.4176 | 921.5246 |
| 2.4183 | 241.2153 |
| 2.435 | 655.3502 |
| 2.4412 | 669.8644 |
| 2.4422 | 562.3495 |
| 2.4471 | 661.3162 |
| 2.4568 | 345.2312 |
| 2.4582 | 981.5237 |
| 2.4582 | 760.646 |
| 2.4583 | 935.5065 |
| 2.46 | 965.5496 |
| 2.4643 | 966.5491 |
| 2.4857 | 516.2822 |
| 2.4861 | 314.1929 |
| 2.494 | 494.2691 |
| 2.4972 | 537.3405 |
| 2.5 | 947.5416 |
| 2.5002 | 759.4352 |
| 2.5017 | 705.3423 |
| 2.5182 | 220.1484 |
| 2.5238 | 941.5797 |
| 2.5276 | 287.1843 |
| 2.5339 | 699.3765 |
| 2.5373 | 176.125 |
| 2.5375 | 535.3822 |
| 2.5429 | 867.4377 |
| 2.5471 | 717.3875 |
| 2.5474 | 538.2938 |
| 2.5518 | 803.4607 |
| 2.5526 | 551.3474 |
| 2.5563 | 328.2014 |
| 2.5564 | 629.3024 |
| 2.5647 | 749.3677 |
| 2.5657 | 560.3078 |
| 2.5665 | 619.3366 |
| 2.5682 | 249.1717 |
| 2.5723 | 613.3276 |
| 2.5729 | 985.6087 |
| 2.5737 | 743.4015 |
| 2.5854 | 555.3482 |
| 2.5884 | 209.145 |
| 2.6084 | 793.3968 |
| 2.6099 | 194.0994 |
| 2.6172 | 781.3097 |
| 2.621 | 995.2926 |
| 2.6237 | 787.4278 |
| 2.6588 | 837.4225 |
| 2.6624 | 604.3305 |
| 2.6719 | 337.2302 |
| 2.6724 | 831.4565 |
| 2.6781 | 223.7796 |
| 2.6889 | 473.1978 |
| 2.6933 | 471.3518 |
| 2.6975 | 73.7956 |
| 2.6981 | 73.6921 |
| 2.7025 | 881.4465 |
| 2.716 | 875.4821 |
| 2.7246 | 326.0914 |
| 2.7315 | 341.2919 |
| 2.7331 | 648.3548 |
| 2.7455 | 925.4746 |
| 2.7544 | 430.331 |
| 2.7575 | 919.5081 |
| 2.7597 | 914.5548 |
| 2.7708 | 401.2527 |
| 2.799 | 963.5311 |
| 2.8232 | 631.499 |
| 2.8587 | 663.7508 |
| 2.8846 | 561.3621 |
| 2.923 | 421.2128 |
| 2.9314 | 448.3163 |
| 2.9497 | 910.4617 |
| 2.9511 | 281.1969 |
| 2.9983 | 279.2154 |
| 3.036 | 435.2866 |
| 3.0603 | 249.1373 |
| 3.0645 | 352.249 |
| 3.0736 | 904.5482 |
| 3.0971 | 388.2269 |
| 3.1012 | 366.2423 |
| 3.1028 | 462.3324 |
| 3.121 | 265.2007 |
| 3.1358 | 406.3094 |
| 3.1603 | 382.2028 |
| 3.2018 | 506.2287 |
| 3.2215 | 396.2643 |
| 3.2387 | 299.1595 |
| 3.2749 | 349.2532 |
| 3.3353 | 383.235 |
| 3.34 | 476.3475 |
| 3.4929 | 416.2827 |
| 3.5418 | 419.2899 |
| 3.5904 | 311.2546 |
| 3.6039 | 426.3118 |
| 3.6161 | 384.1805 |
| 3.6347 | 363.2723 |
| 3.71 | 462.2396 |
| 3.822 | 375.3251 |
| 3.9018 | 524.359 |
| 3.903 | 552.2559 |
| 3.9048 | 624.3012 |
| 3.9056 | 523.1987 |
| 3.9062 | 659.3124 |
| 3.9065 | 512.2056 |
| 3.9071 | 360.128 |
| 3.9072 | 507.2279 |
| 3.9076 | 179.1016 |
| 3.9085 | 633.2545 |
| 3.9087 | 407.2023 |
| 3.9097 | 811.3974 |
| 3.9102 | 235.1543 |
| 3.9107 | 194.1334 |
| 3.9123 | 340.219 |
| 3.9567 | 331.2544 |
| 4.0569 | 614.414 |
| 4.0889 | 427.2668 |
| 4.1585 | 412.2097 |
| 4.4507 | 313.2471 |
| 4.492 | 213.21 |
| 4.7564 | 419.3297 |
| 4.8684 | 316.3267 |
| 4.9231 | 679.3958 |
| 5.0309 | 371.3298 |
| 5.0669 | 249.1721 |
| 5.113 | 288.2617 |
| 5.2412 | 359.2446 |
| 5.2416 | 354.2888 |
| 5.9804 | 397.2859 |
| 6.0007 | 704.4954 |
| 6.1099 | 663.4365 |
| 6.1611 | 358.2048 |
| 6.3103 | 324.225 |
| 6.4477 | 762.5419 |
| 6.4996 | 380.3362 |
| 7.2067 | 468.4059 |
| 7.8775 | 694.6337 |
| 7.978 | 519.369 |
| 8.0654 | 536.1726 |
| 8.242 | 480.3887 |
| 8.2803 | 265.1995 |
| 8.3096 | 586.3744 |
| 8.3298 | 595.4725 |
| 8.3541 | 628.1956 |
| 8.3683 | 920.6875 |
| 8.4566 | 666.3712 |
| 8.4631 | 638.3375 |
| 8.5118 | 735.4647 |
| 8.6383 | 395.3027 |
| 8.6839 | 478.4053 |
| 8.7214 | 697.4498 |
| 8.9295 | 492.3757 |
| 8.9616 | 265.1994 |
| 9.0079 | 816.5895 |
| 9.0103 | 832.2397 |
| 9.0656 | 656.5107 |
| 9.0977 | 889.597 |
| 9.2098 | 447.3458 |
| 9.3556 | 907.2602 |
| 9.3823 | 526.4455 |
| 9.3963 | 609.4835 |
| 9.4172 | 516.405 |
| 9.8199 | 694.5819 |
| 9.8647 | 611.531 |
| 9.8826 | 601.4429 |
